# Development and evaluation of a multi-target droplet digital PCR assay for highly sensitive and specific detection of *Yersinia pestis*

**DOI:** 10.1371/journal.pntd.0012167

**Published:** 2024-05-03

**Authors:** Yanting Zhao, Ziheng Yan, Kai Song, Yanbing Li, Leiming Shen, Yiming Cui, Zongmin Du, Ruifu Yang, Yajun Song, Lan Jing, Yong Zhao

**Affiliations:** 1 College of Horticulture and Plant Protection, Inner Mongolia Agricultural University, Hohhot, Inner Mongolia, China; 2 State Key Laboratory of Pathogen and Biosecurity, Beijing Institute of Microbiology and Epidemiology, Beijing, China; 3 Department of Laboratory Medicine, Xiangya Hospital of Central South University, Changsha, China; 4 Beijing Key Laboratory of POCT for Bioemergency and Clinic, Beijing, China; University of Texas Medical Branch, UNITED STATES

## Abstract

**Background:**

Plague, caused by the bacterium *Yersinia pestis*, is a zoonotic disease that poses considerable threats to human health. Nucleic acid tests are crucial for plague surveillance and the rapid detection of *Y*. *pestis*. However, inhibitors in complex samples such as soil and animal tissues often hamper nucleic acid detection, leading to a reduced rate of identifying low concentrations of *Y*. *pestis*. To address this challenge, we developed a sensitive and specific droplet digital polymerase chain reaction (ddPCR) assay for detecting *Y*. *pestis* DNA from soil and animal tissue samples.

**Methods:**

Three genes (*ypo2088*, *caf1*, and *pla*) from *Y*. *pestis* were used to develop a multi-target ddPCR assay. The limits of detection (LoD), reproducibility, and specificity were assessed for bacterial genomic DNA samples. The ability of the assay to detect low concentrations of *Y*. *pestis* DNA from simulated soil and mouse liver tissue samples was respectively evaluated and compared with that of quantitative real-time PCR (qPCR).

**Results:**

The results showed that the ddPCR LoDs ranged from 6.2 to 15.4 copies/reaction for the target genes, with good reproducibility and high specificity for *Y*. *pestis*. By testing 130 soil and mouse liver tissue samples spiked with *Y*. *pestis*, the ddPCR assay exhibited a better sensitivity than that of the qPCR assay used in the study, with LoDs of 10^2^ colony forming units (CFU)/100 mg soil and 10^3^ CFU/20 mg liver. Moreover, the assay presented good quantitative linearity (R^2^ = 0.99) for *Y*. *pestis* at 10^3^–10^6^ CFU/sample for soil and liver samples.

**Conclusion:**

The ddPCR assay presented good performance for detecting *Y*. *pestis* DNA from soil and mouse tissue samples, showing great potential for improving the detection rate of low concentrations of *Y*. *pestis* in plague surveillance and facilitating the early diagnosis of plague cases.

## Author summary

*Yersinia pestis* is the causative agent of three historic plague pandemics. Presently, the plague remains endemic or sporadic in many countries. The wide distribution of natural plague foci in Asia, Africa, and the Americas reminds us of the threat of a plague outbreak. The timely detection and surveillance of *Y*. *pestis* prevalence in animals and the environment in natural epidemic foci is essential for preventing and controlling plague outbreaks. In this study, we developed a sensitive and specific ddPCR assay for detecting low concentrations of *Y*. *pestis* DNA from soil and mouse tissue samples. The ddPCR assay presented a considerable improvement in sensitivity compared to the qPCR assay used in the study, thus providing a promising tool for detecting *Y*. *pestis* DNA from complex samples and facilitating the accurate diagnosis of plague cases.

## Introduction

Plague is a zoonotic disease spread by the bacterium *Yersinia pestis* through rodent fleas [1]. Human infection with *Y*. *pestis* usually occurs via direct contact with infected animals or through flea bites [2]. Three major plague pandemics in history have caused millions of human deaths [3]. The 2017 plague epidemic in Madagascar resulted in more than 2,400 plague cases and hundreds of deaths, indicating that the plague remains a major public health concern [4–6]. Early detection and surveillance of *Y*. *pestis* prevalence in animals and the environment in natural epidemic foci is essential for preventing and controlling plague outbreaks [7].

The polymerase chain reaction (PCR) is widely used for the rapid and sensitive detection of *Y*. *pestis* [4, 8]. Most methods select specific genes located on virulence plasmids of *Y*. *pestis* as detection targets, such as the *caf1* gene on the pMT1 plasmid and the *pla* gene on the pPCP1 plasmid [9–11]. However, this strategy carries a risk of false negatives because the corresponding plasmids may be lost from *Y*. *pestis* cells. *Y*. *pestis* strains lacking the pPCP1 or pMT1 plasmid have been isolated in nature [12, 13]. To address this issue, researchers have developed detection methods targeting chromosome-encoded genes specific to *Y*. *pestis* [14, 15]. In 2021, the World Health Organization (WHO) revised the international definition of plague cases and recommended a combination of at least two different gene targets (including *pla*, *caf1*, *ypo2088*, and *ypo2486*) to detect *Y*. *pestis* by PCR-based methods; two of these genes must be positive for the laboratory confirmation of *Y*. *pestis* [16].

In general, the most common sample types encountered in plague surveillance are animal organs or tissues and contaminated environmental samples. These samples usually contain many PCR inhibitors (such as humic acid, heparin, and hemoglobin), which may affect the detection performance of PCR [17]. In our previous study [18], we established a recombinase polymerase amplification assay coupled with the clustered regularly interspaced short palindromic repeats technique (RPA-CRISPR) to detect *Y*. *pestis* and evaluated its sample tolerance to soil, mouse blood, and mouse lung tissue. The detection sensitivity of tissue samples (10^5^ colony forming units (CFU)/20 mg mouse lung tissue) was significantly decreased compared with that of soil samples (10^3^ CFU/100 mg soil) and blood samples (10^2^ CFU/100 μL mouse blood). We hypothesized that this reduction in sensitivity may be due to the inhibitors (such as heparin) from tissue samples that remain in the DNA extract.

As a new generation of PCR technology, droplet digital PCR (ddPCR) possesses the advantages of absolute quantification, high sensitivity, and strong sample tolerance over traditional PCR methods [19, 20]. In the ddPCR reaction, the sample is partitioned into over ten thousand separate droplets, and the fluorescence signal of each droplet is collected after PCR amplification. These individual droplets mitigate the influence of inhibitors on PCR amplification, as the positive signal can be still retained even if moderate PCR inhibition is present in the droplet [21, 22]. Therefore, ddPCR is suitable for highly sensitive and specific *Y*. *pestis* detection in complex sample matrices. In a previous study [23], researchers developed a ddPCR detection method for *Y*. *pestis* with a high sensitivity of ~10^2^ CFU/100 mg soil. However, this assay only targeted one gene on the *Y*. *pestis* chromosome, which did not meet the latest WHO recommended guidelines for the plague detection.

In this study, we established a multi-target ddPCR assay to detect *Y*. *pestis* DNA using three target genes (*ypo2088*, *caf1*, and *pla*). The reaction can be performed in a single tube and requires a digital PCR instrument with two-color fluorescent channels. The performance of the assay was comprehensively evaluated, including detection sensitivity, reproducibility, quantitative accuracy, and specificity. Moreover, the assay’s ability to detect low concentrations of *Y*. *pestis* DNA from soil and mouse liver tissue samples was assessed and compared with that of quantitative real-time PCR (qPCR).

## Methods

### DNA extraction

*Y*. *pestis* strain 201, harboring four plasmids (pPCP1, pCD1, pMT1, and pCRY), was used in the study. This strain belongs to the biovar Microtus and is highly virulent in mice but avirulent in humans [24, 25]. The bacteria were inoculated in 5 mL of Luria-Bertani (LB) medium at 26°C for about 10 hours, with shaking at 200 r/min.

500 μL *Y*. *pestis* culture was centrifuged at 8,000 × *g* for 5 min to collect the bacterial pellet. DNA was then extracted using the QIAamp DNA Mini kit (51304) (Qiagen, Germany) according to the manufacturer’s instructions. Finally, DNA was eluted with 100 μL double distilled water (ddH_2_O). The DNA concentration was measured using a NanoDrop 2000 spectrophotometer (Thermo Fisher Scientific, USA).

### Primers and probes

The primers and TaqMan probes for *ypo2088* were obtained according to a previous report [26]. The primers and TaqMan probes for the *caf1* and *pla* genes were designed using the Primer Express software version 3.0 (Thermo Fisher Scientific). The sequences are shown in Table 1. Sangon Biotech (Shanghai, China) synthesized all primers and probes.

**Table 1:** Primers and probes for detecting *Y*. *pestis*.

Target gene	Oligo name	Sequence 5’ to 3’	Amplicon size (bp)
***ypo2088***	*ypo2088*-F	GGACGGCATCACGATTCTCT	67
	*ypo2088*-R	CCTGAAAACTTGGCAGCAGTT	
	*ypo2088*-T	HEX-CCCTCGAATCGCTGGCAGCAACTG-BHQ1	
***caf1***	*caf1*-F	CAAGCACCACTGCAACGG	133
	*caf1*-R	CCAAGAGTAAGCGTACCAACAAGTA	
	*caf1*-T	HEX-ATGACGTCGTCTTGGCTACGGG-BHQ1	
***pla***	*pla*-F	GCAGCATCATCTCAGTTAATACCAA	107
	*pla*-R	TCTGCGTCATAAAGCATTTCATG	
	*pla*-T	FAM-TGCAGCCTCCACCGGGATGC-BHQ1	

### ddPCR assay

For each single-target gene ddPCR assay, the reaction mixture (20 μL) contained 10 μL Supermix for probes (Bio-Rad, USA), 900 nM forward primer, 900 nM reverse primer, 100–1500 nM TaqMan probe, 2 μL DNA template, and ddH_2_O.

For the multiplex ddPCR assay, the reaction mixture (20 μL) contained 10 μL Supermix for Probes (No dUTP) (Bio-Rad), 900 nM each forward primer (*ypo2088*-F, *caf1*-F, and *pla*-F), 900 nM each reverse primer (*ypo2088*-R, *caf1*-R, and *pla*-R), 250 nM *pla*-T, 250 nM *caf1*-T, 500 nM *ypo2088*-T, 2 μL DNA template, and ddH_2_O.

Next, 20 μL ddPCR reaction mixture and 70 μL Droplet generation Oil For Probe (Bio-Rad) were used to generate droplets with the QX200 ddPCR system (Bio-Rad). The droplets were amplified using a T100 Thermal Cycler (Bio-Rad) under the following conditions: initial denaturation at 95°C for 10 min, 40 cycles of denaturation (94°C, 0.5 min), and annealing/extension (60°C, 1 min), enzyme deactivation (98°C, 10 min), and incubation at 12°C for 30 min. After amplification, the products were detected and analyzed with a droplet reader in the QX200 ddPCR system.

### Real-time PCR assay

For single-target qPCR, the reaction mixture (20 μL) contained 10 μL qPCR Probe SuperMix (Takara, Japan), 400 nM forward primer, 400 nM reverse primer, 200 nM probe, 2 μL DNA template, and ddH_2_O. The primer and probe sequences were the same as those listed in Table 1.

Real-time PCR was performed on a CFX96 Real-Time System (Bio-Rad) under the following conditions: 95°C for 5 min, followed by 40 cycles of 95°C for 10 s and 60°C for 60 s. The cycle threshold (Ct) was determined using the single threshold method provided in the Bio-Rad CFX Maestro software (Bio-Rad).

### Evaluation of the ddPCR assay using diluted DNA samples

The DNA templates were diluted to concentrations ranging from 1 ng/μL to 1 fg/μL and were detected using the ddPCR assay as described above. According to the Clinical and Laboratory Standards Institute (CLSI) EP17-A2 guideline, the limit of blank (LoB) for each target gene was calculated by testing 35 blank samples (ddH_2_O) (details in S1 Table). A ddPCR readout above the LoB value is considered positive. The lowest DNA concentration that could be consistently detected (n = 3) was the limit of detection (LoD). The coefficient of variation (CV) of the repeated tests was calculated to evaluate reproducibility. The quantification curve was plotted using the logarithm of the genomic equivalent (GE) number of *Y*. *pestis* cells per reaction [Log_10_ (GE /reaction)] as the *x-*axis and the logarithm of the ddPCR readouts [Log_10_ (copy number/reaction)] as the *y-*axis. The coefficient of determination (R^2^) was calculated using Origin software (OriginLab).

### Specificity of the ddPCR assay

DNA samples from seven other bacterial species closely related to *Y*. *pestis* and four other zoonotic pathogens, including *Yersinia kristensenii* (ATCC 33638), *Yersinia enterocolitica* (ATCC 9610), *Yersinia pseudotuberculosis* (ATCC 28933), *Yersinia frederiksenii* (ATCC 33641), *Yersinia rohdei* (ATCC 43380), *Yersinia ruckeri* (ATCC 29473), *Yersinia mollaretii* (ATCC 43969), *Bacillus anthracis* (CBSLAM 00067), *Brucella abortus* (CBSLAM 6148), *Burkholderia pseudomallei* (ATCC 23343), and *Francisella tularensis* (CBSLAM 6339) were used to assess the specificity of the ddPCR assay. Additionally, DNA samples from three plasmid-cured *Y*. *pestis* strains, including *Y*. *pestis* (deletion of pMT1), *Y*. *pestis* (deletion of pPCP1), and *Y*. *pestis* (deletion of pMT1 and pPCP1), were used to assess the inclusivity of the ddPCR assay. The plasmid deletion method was described in our previous work [27]. All DNA samples (0.1 ng/μL) were detected by the ddPCR assay as described above. Samples were considered positive for *Y*. *pestis* when at least two of the three target genes of *Y*. *pestis* were detected.

### Detection of *Y*. *pestis* DNA from pure bacterial cultures

*Y*. *pestis* cells were washed three times and resuspended in sterile normal saline (NS) solution (0.9% NaCl). The bacterial number was determined with the plate counting method. Serial dilutions of *Y*. *pestis* in NS solutions were then prepared from ~10 CFU/mL to 10^8^ CFU/mL. For each concentration, 0.5 mL bacterial suspension was subjected to DNA extraction using the QIAamp DNA Mini kit (51304) (Qiagen, Germany) as described above. The extracted DNA was eluted with 50 μL ddH_2_O. Finally, 2 μL DNA was used as the template and detected with the ddPCR and qPCR methods, respectively. The NS solution was used as the negative control (NC). Each test was repeated in triplicate. The LoB was obtained by testing the NS solutions without spiking *Y*. *pestis*.

### Detection of *Y*. *pestis* DNA from simulated soil samples

Loam soil samples were collected from a flower bed in local outdoor environment and equally divided into 65 portions of 250 mg each. Then, 100 μL NS solution containing *Y*. *pestis* cells was added to 35 of the samples to prepare soil samples spiked with different concentrations of *Y*. *pestis* (10–10^6^ CFU/sample, five replicates for each concentration). 100 μL sterile NS solution was added to another 30 samples as NCs. According to the manufacturer’s instructions, DNA was extracted using the TIANamp Soil DNA Kit (TIANGEN Biotech, China). Finally, the DNA was eluted with 50 μL ddH_2_O, and 2 μL was used as the template for the ddPCR and qPCR assays, as mentioned above. The corresponding LoB was obtained by testing soil samples spiked without *Y*. *pestis*.

### Detection of *Y*. *pestis* DNA from simulated liver samples

A batch of mouse liver homogenate (200 mg/mL in NS solution) was divided into 65 portions (100 μL, 20 mg tissue). Then, 100 μL NS solution containing *Y*. *pestis* cells was added to 35 of the samples, resulting in *Y*. *pestis*-spiked liver tissue samples containing bacteria ranging from 10 to 10^6^ CFU/sample (five replicates for each concentration). An additional 30 samples were supplemented with 100 μL sterile NS solution as NCs. DNA was extracted using the DNeasy Blood & Tissue Kit (69504) (Qiagen, Germany) according to the manufacturer’s instructions. Finally, the DNA was eluted with 50 μL ddH_2_O, and 2 μL was used as the template for the ddPCR and qPCR assays. The corresponding LoB was obtained by testing tissue samples spiked without *Y*. *pestis*.

## Results

### Establishment of the multi-target ddPCR assay for *Y*. *pestis* detection

**Fig 1:**
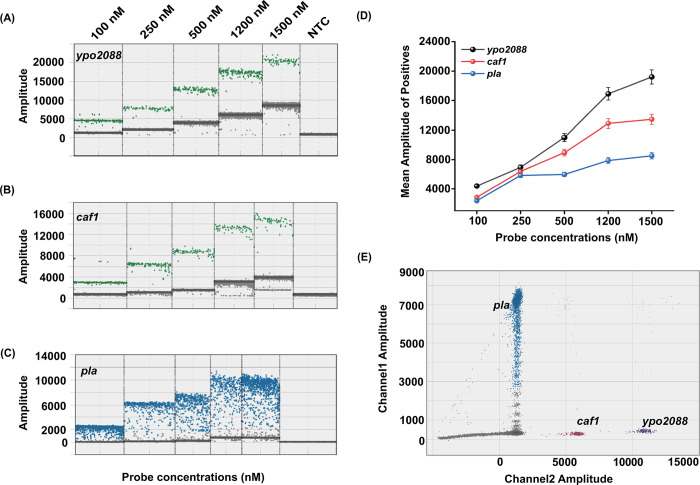
Establishment of the multi-target ddPCR assay. **(A–C)** Amplification plots and **(D)** comparisons of the fluorescence amplitude of single-target ddPCR mixtures containing probes of different concentrations. **(E)** Two-dimensional fluorescence scatter plot (2D plot) of the ddPCR assay for the simultaneous detection of three different gene targets. The *x-*axis represents the fluorescence amplitude in the FAM channel, and the *y*-axis represents the fluorescence amplitude in the HEX channel.

We established three single-target ddPCR reactions for the *ypo2088*, *caf1*, and *pla* genes of *Y*. *pestis*. The fluorescence amplitude of positive droplets gradually increased with increasing probe concentration from 100 to 1500 nM (Fig 1). Among the three targets, the amplification results of the *pla* gene presented the “rain” phenomenon (a subset of the droplets exhibits a faint fluorescence intensity, making the delineation between positive and negative droplets not clear) [28, 29], although we performed multiple optimizations (such as adjusting the annealing temperature, the number of amplification cycles, and the temperature ramp rate in the cycling). To avoid possible interference, the labeling fluorescence of the *pla* probe was designed to be different from that of the *ypo2088* and *caf1* probes when establishing the multiplex ddPCR assay. To detect *ypo2088* and *caf1* simultaneously in one channel, we adopted the “amplitude-based multiplex strategy”, where the final probe concentrations of *ypo2088* and *caf1* were 500 nM and 250 nM, respectively. Under these conditions, the difference in fluorescence amplitudes between *ypo2088* and *caf1* is significant enough to distinguish the two targets.

Finally, the multi-target ddPCR assay for *Y*. *pestis* detection was optimized as follows: the *pla* probe was labeled with FAM at a final concentration of 250 nM; the *caf1* and *ypo2088* probes were both labeled with HEX at a final concentration of 250 nM and 500 nM, respectively. After optimization, the multi-target ddPCR assay could simultaneously detect three gene targets of *Y*. *pestis* in a single tube with a common two-color ddPCR instrument.

### Sensitivity, linearity, and reproducibility of the ddPCR assay

**Fig 2:**
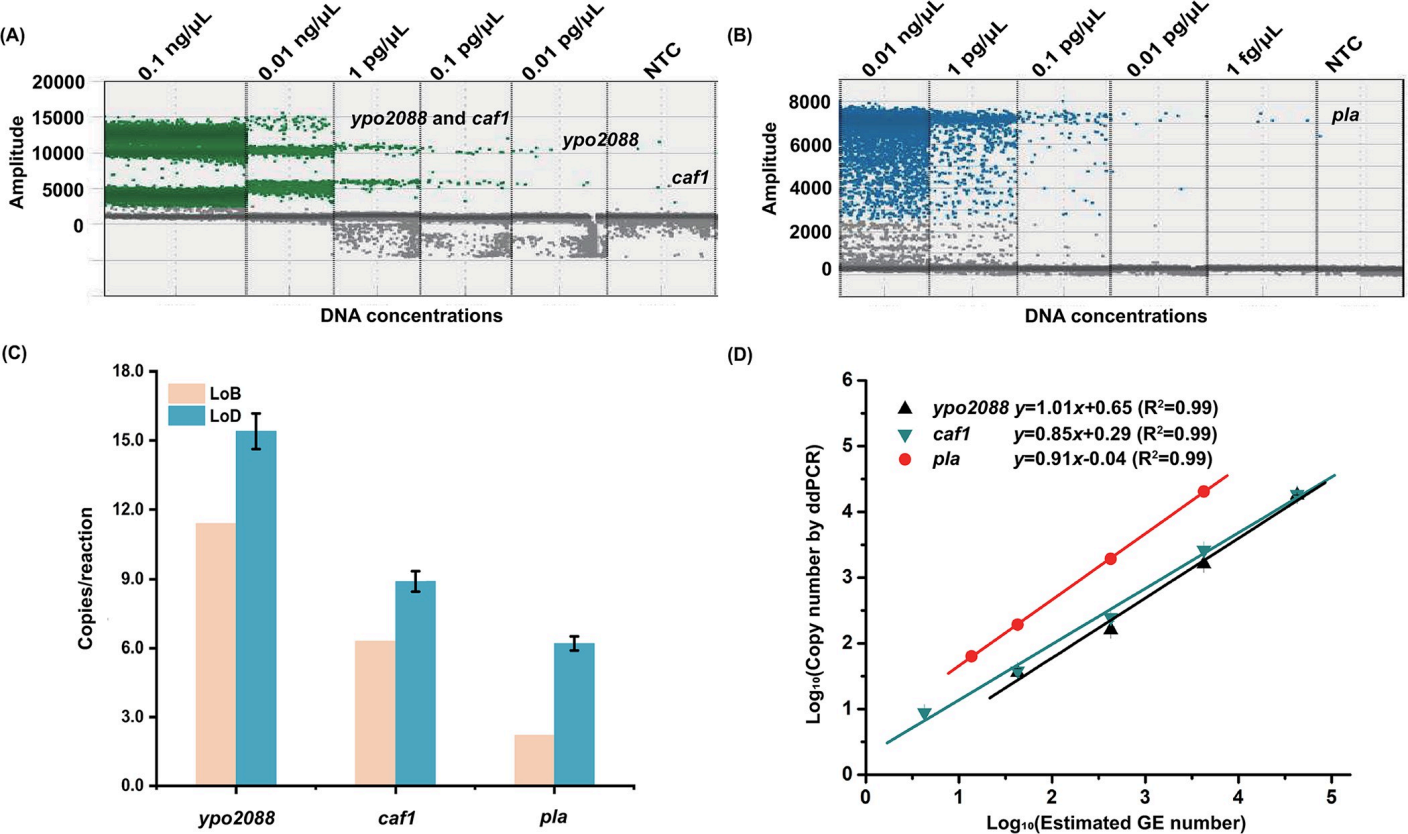
Performance of the multi-target ddPCR assay for detecting diluted genomic DNA samples of *Y*. *pestis*. **(A, B)** Amplification plots of the *ypo2088*, *caf1*, and *pla* genes by ddPCR. The grey dots in the graph refer to the droplets containing no target gene. The green dots represent the droplets containing either *ypo2088* or *caf1* or both genes, as detailed in the graph. The blue dots represent droplets containing the *pla* gene. **(C)** LoB and LoD values of the three gene targets. **(D)** Quantification linearity of the assay targeting different genes. The *x-*axis refers to Log_10_ (estimated GE number/reaction), and the *y-*axis refers to Log_10_ (copy number/reaction) by ddPCR. Each test was repeated in triplicate.

By testing 35 blank samples (ddH_2_O), the LoBs for *ypo2088*, *caf1*, and *pla* were determined to be 11.4, 6.4, and 2.2 copies/reaction, respectively (S1 Table). Then, serially diluted *Y*. *pestis* genomic DNA solutions were tested in triplicate to assess detection sensitivity (Fig 2A and 2B). The results shows that the LoDs for *ypo2088* and *caf1* were 15.4 and 8.9 copies/reaction, respectively; while the LoD for *pla* was the lowest, reaching 6.2 copies/reaction (Fig 2C). The ddPCR assay also shows a good linearity (R^2^ = 0.99) for quantifying these three gene targets, with dynamic ranges spanning four orders of magnitude (Fig 2D). In the dynamic range, all intra-assay CV values were within 15% (S1 Data), demonstrating good test reproducibility. The *x*-axis unit in Fig 2D, genomic equivalent (GE) number of *Y*. *pestis* cells per reaction, were estimated by the amount of genomic DNA template (pg) and the genome mass of *Y*. *pestis* (213 GEs/pg) [10]. The copy numbers of *ypo2088* and *caf1* determined by ddPCR were more consistent with the estimated GE number, whereas the copy number of *pla* was approximately 10-fold higher than the GE number. This result is reasonable because the pPCP1 plasmid that harbors the *pla* gene is multi-copy in *Y*. *pestis* [30].

### Specificity of the ddPCR assay

Genomic DNA (0.1 ng/μL) samples, from seven other bacterial species within the *Yersinia* genus and four other zoonotic pathogens, were tested using the developed ddPCR assay for *Y*. *pestis* detection. According to the results (Table 2), no positive droplets were observed for *ypo2088*, *caf1*, and *pla* genes in these eleven bacteria, Additionally, *Y*. *pestis* strains lacking the pMT1 or pPCP1 plasmid could still be detected and identified as *Y*. *pestis* by the ddPCR assay. However, for strains that were missing both pMT1 and pPCP1 plasmids, the assay failed as expected because only the chromosomal gene *ypo2088* could be detected. For this assay, samples were considered positive for *Y*. *pestis* when at least two of the three target genes of *Y*. *pestis* were detected.

**Table 2:** Specificity evaluation of the ddPCR assay.

Bacteria	ddPCR readout (copies/reaction)		
	*ypo2088*	*caf1*	*pla*
***Y*. *kristensenii***	0.00 (-)	0.80 (-)	0.80 (-)
***Y*. *enterocolitica***	0.05 (-)	1.20 (-)	0.60 (-)
***Y*. *pseudotuberculosis***	0.04 (-)	2.00 (-)	0.40 (-)
***Y*. *frederiksenii***	0.80 (-)	1.00 (-)	0.60 (-)
***Y*. *rohdei***	0.00 (-)	0.40 (-)	0.00 (-)
***Y*. *ruckeri***	0.00 (-)	2.00 (-)	0.00 (-)
***Y*. *mollaretii***	0.00 (-)	1.20 (-)	0.60 (-)
***F*. *tularensis***	0.00 (-)	2.00 (-)	0.40 (-)
***B*. *abortus***	0.00 (-)	2.60 (-)	0.60 (-)
***B*. *pseudomallei***	0.00 (-)	2.00 (-)	0.40 (-)
***B*. *anthracis***	0.00 (-)	3.20 (-)	1.20 (-)
***Y*. *pestis* (pPCP1** ^**-**^ **)**	29992.20 (+)	33506.80 (+)	1.00 (-)
***Y*. *pestis* (pMT1** ^**-**^ **)**	14559.60 (+)	2.80 (-)	84336.40 (+)
***Y*. *pestis* (pPCP1** ^**-**^ **, pMT1** ^**-**^ **)**	20204.20 (+)	2.40 (-)	1.00 (-)
***Y*. *pestis***	11489.00 (+)	13330.80 (+)	70874.40 (+)

Data are presented as averages (n = 3).

### Detection of *Y*. *pestis* DNA from pure bacterial cultures by ddPCR and qPCR

**Fig 3:**
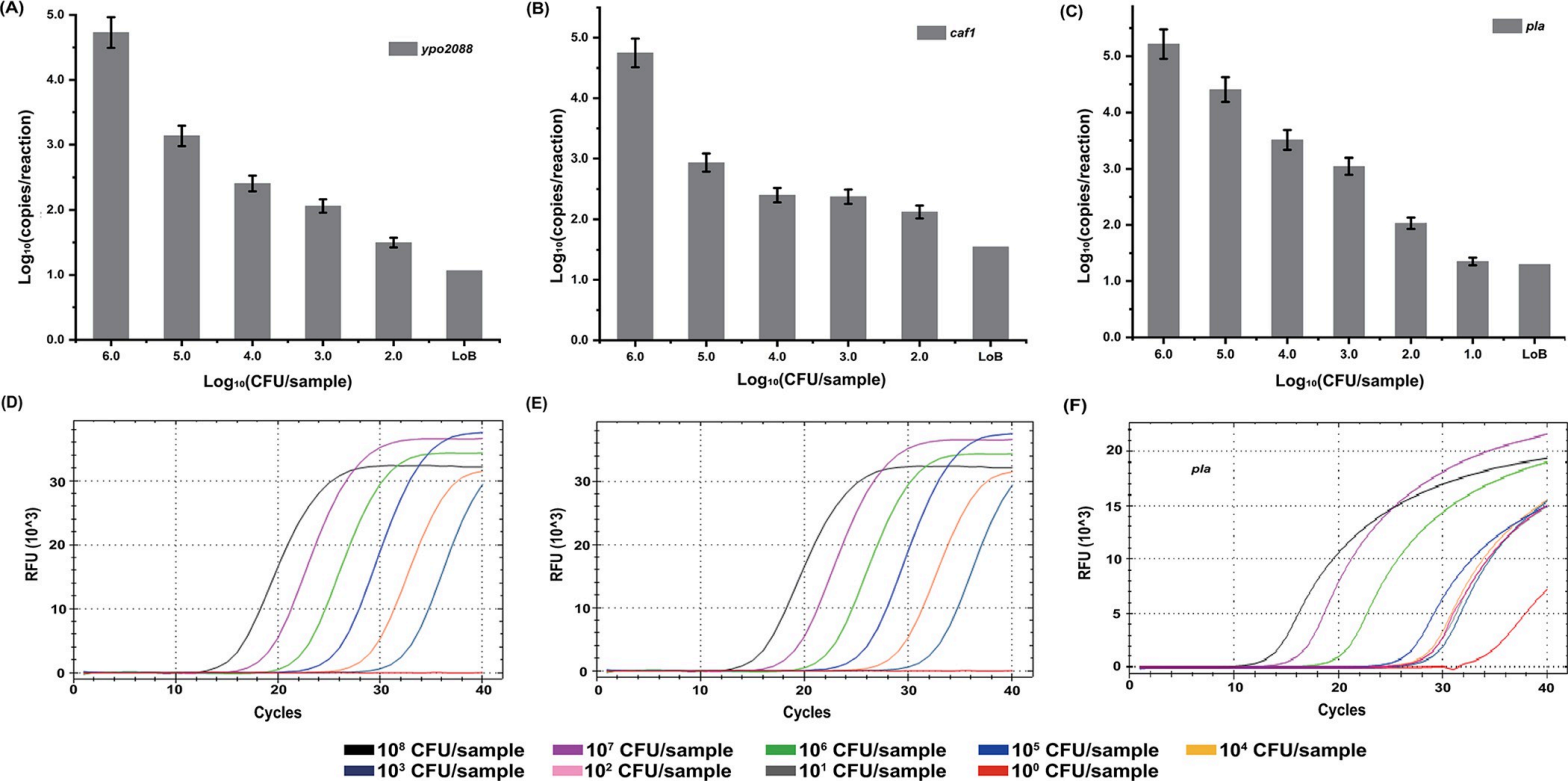
Performance of the multi-target ddPCR and single-target qPCR assays in detecting genomic DNA samples of *Y*. *pestis*. (**A–C**) Serial dilutions of *Y*. *pestis* ranging from 10^2^ to 10^6^ CFU/mL were subjected to DNA extraction and ddPCR detection. The *x-*axis refers to the bacterial concentration [Log_10_ (CFU/sample]), and the *y-*axis refers to the ddPCR readout [Log_10_ (copies/reaction)]. (**D–F**) The same batch of DNA samples were also tested by single-target qPCR assays. Each test was repeated in triplicate.

Genomic DNA was extracted from serial dilutions (10^1^–10^8^ CFU/sample) of pure *Y*. *pestis* cultures and detected with ddPCR. As shown in Fig 3A–3C, the detection sensitivity of *Y*. *pestis* was 10^2^ CFU/sample (1 mL NS buffer) when targeting *ypo2088* or *caf1*, whereas it was 10^1^ CFU/sample when targeting the *pla* gene. Consequently, the lowest bacterial concentration that could be reliably detected by ddPCR was set at 10^2^ CFU/sample, with at least two gene targets testing positive. The same batch of DNA samples was also subjected to qPCR analysis, which was established for the three genes using the same primer and probe sequences as the ddPCR assay. The detection sensitivity by qPCR was set at 10^3^ CFU/sample, considering that the lowest detectable bacterial concentration when targeting *ypo2088*, *caf1*, and *pla* was 10^3^, 10^3^, and 10 CFU/sample, respectively (Fig 3D–3F). This result demonstrates a 10-fold improvement in the detection sensitivity of ddPCR (10^2^ CFU/sample) over the single-target qPCR (10^3^ CFU/sample) employed in the study.

### Detection of *Y*. *pestis* DNA from simulated environmental and biological samples by ddPCR and qPCR

**Fig 4:**
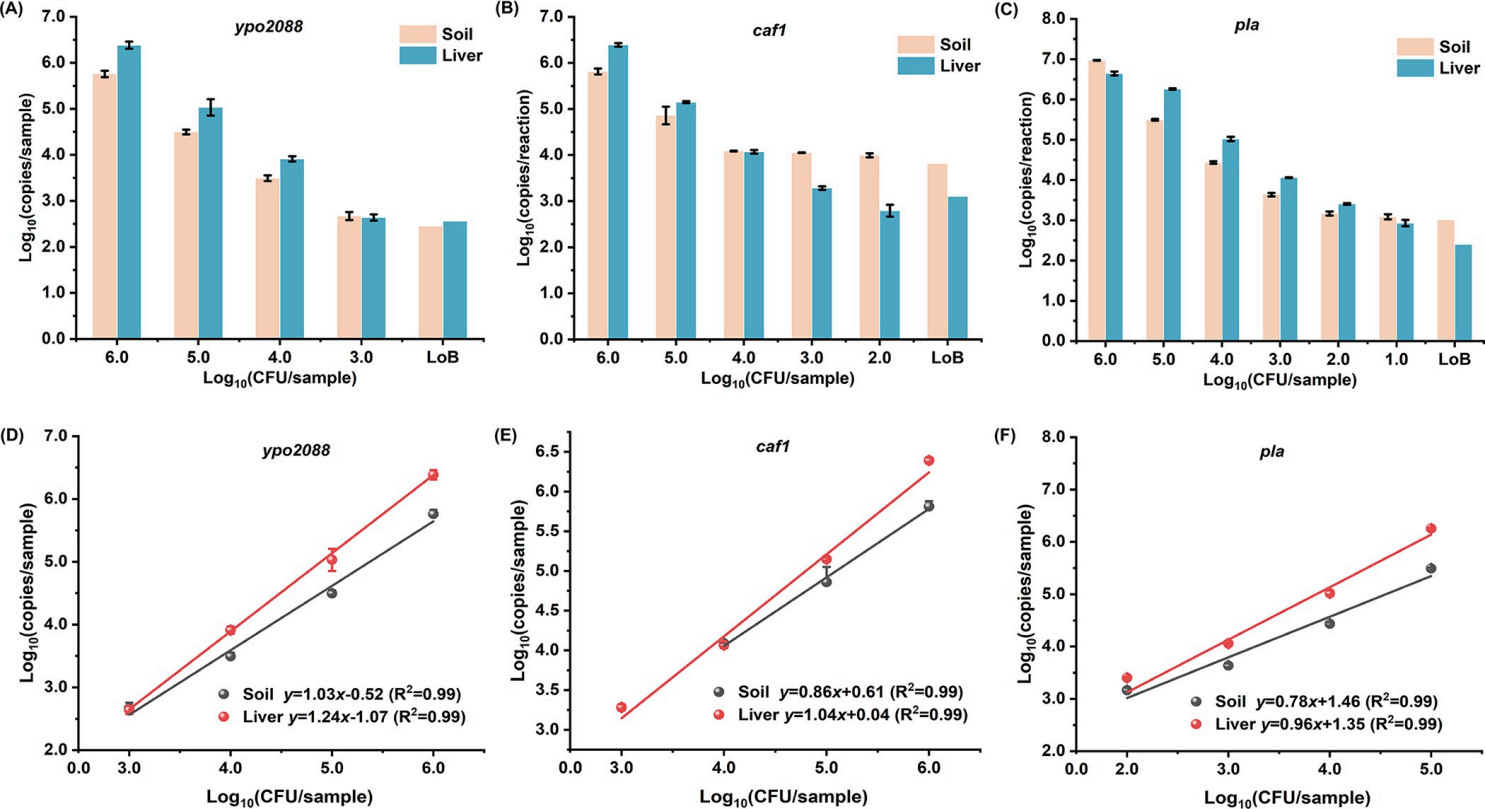
Performance of the ddPCR assay in detecting *Y*. *pestis* DNA from soil and mouse liver tissue samples. **(A–C)** The ddPCR readouts of the detection of *ypo2088*, *caf1*, and *pla*, respectively. **(D**–**F)** Linearity and dynamic ranges of the ddPCR assay for quantifying *Y*. *pestis* in soil and liver tissue samples with *ypo2088*, *caf1*, and *pla* as the target gene, respectively. The *x-*axis refers to the bacterial concentration [Log_10_ (CFU/sample)], and the *y-*axis refers to the ddPCR readout [Log_10_ (copies/reaction)]. Each test was repeated five times.

We further evaluated the performance of the ddPCR assay for detecting *Y*. *pestis* DNA from simulated soil and mouse liver tissue samples, which were artificially contaminated with a serial numbers of *Y*. *pestis* cells. DNA was extracted and analyzed according to the method described above. The lowest amount of *Y*. *pestis* that could be detected in soil sample (100 mg) was 10^3^, 10^2^, and 10 CFU/sample when targeting *ypo2088*, *caf1*, and *pla*, respectively (Fig 4A–4C). For liver tissue sample (20 mg), the lowest amount detected was 10^3^, 10^3^, and 10 CFU/sample, respectively. Consequently, the ddPCR detection sensitivity of *Y*. *pestis* in soil and liver tissue samples was set at 10^2^ and 10^3^ CFU/sample, respectively. In comparison, the single-target qPCR assay exhibited a lower sensitivity (10^4^ CFU/sample) for both soil and liver tissue samples (S1 Fig). Therefore, the ddPCR assay presented a 10–100 times improvement in sensitivity compared to the qPCR assays employed in the study. Given its high sensitivity, the actual positive rate of ddPCR (71.43%, 50/70) was higher than that of qPCR (57.14%, 40/70) when analyzing the combined 130 samples of *Y*. *pestis*-spiked soil and liver tissue samples (S2 and S3 Tables).

Moreover, the ddPCR sensitivity for soil samples remained consistent with that of pure *Y*. *pestis* cultures (10^2^ CFU/sample), demonstrating a good tolerance to soil. Although the sensitivity for liver tissue sample was reduced by 10 times, it was still better than that of qPCR. Furthermore, the quantitative accuracy of ddPCR was not significantly affected by the soil and tissue samples (Fig 4D–4F), which exhibited a good linearity (R^2^ = 0.99) and quantitative range for *Y*. *pestis* at concentrations from 10^3^ to 10^6^ CFU/sample when targeting the *ypo2088* gene.

## Discussion

In this study, we established a multi-target ddPCR assay for the simultaneous detection of three specific genes of *Y*. *pestis*, including *ypo2088* (on the chromosome), *caf1* (on the pMT1 plasmid), and *pla* (on the pPCP1 plasmid). For this assay, at least two positive gene detections are required to call a positive detection of *Y*. *pestis*, in order to enhance the assay specificity and avoid false negatives caused by plasmid deficiency. The performance of the assay was comprehensively evaluated; it exhibited low LoDs ranging from 6.2 to 15.4 copies/reaction for the targeted genes and displayed high specificity in identifying *Y*. *pestis*. Furthermore, its capacity to detect low concentrations of *Y*. *pestis* (10^2^–10^3^ CFU/sample) DNA from soil and mouse liver tissue samples was also demonstrated, revealing a 10–100 times advantage in sensitivity over the qPCR method (10^4^ CFU/sample) employed in the study.

For detecting *Y*. *pestis*, *pla* is the most sensitive gene marker among the three gene targets because the pPCP1 plasmid that encodes *pla* is multi-copy in *Y*. *pestis* [30]. However, the detection of *Y*. *pestis* using only *pla* gene as the target is not reliable because this gene is not specific enough for *Y*. *pestis* [4, 31]. The presence of *pla* sequences has been found in other bacterial strains such as *Amphibacillus jilinensis* Y1 (100% similarity with the *pla* gene sequences from *Y*. *pestis* CO92, 939/939 identities), *Citrobacter koseri* (98.7% similarity, 927/939 identities), and *Escherichia coli* strain FHI29 (98.5% similarity, 925/939 identities). Consequently, the results for *Y*. *pestis* detection must be reported with caution when only *pla* gene is positive in samples. Considering that the *pla* gene encodes an important virulence factor (plasminogen activator) for *Y*. *pestis*, we still included this gene target in our ddPCR assay; at the same time, other two specific gene targets (*ypo2088* and *caf1*) were included to ensure high specificity to *Y*. *pestis*.

Compared with earlier reported ddPCR and qPCR assays for *Y*. *pestis* detection, our assay provides the advantage of both comprehensive gene targets and high detection sensitivity (Table 3). The qPCR-based FilmArray Biothreat Panel test and GeneXpert tularensis-anthracis-pestis (TAP) assay [9, 10] facilitate the sample-to-answer detection of *Y*. *pestis*; however, their target genes were only on the plasmids (pMT1 or/and pPCP1). The TaqMan Array Card (TAC) assay [11] developed for *Y*. *pestis* selects three distinctive gene targets and its sensitivity could reach 10^3^ CFU/sample for mouse blood samples. Specifically, the above three qPCR-based assays for *Y*. *pestis* all pertain to blood samples and do not involve sensitivity evaluations for soil or rodent organ samples. Qu *et al*. [26] established two single-target qPCR assays (targeting the 3a sequence on the chromosome and the *caf1* gene, respectively) and demonstrated a sensitivity of 10^4^ CFU/200 mg soil for the detection of *Y*. *pestis* in soil samples. In contrast to this report, our ddPCR assay demonstrates an approximately 100-fold improvement in sensitivity, allowing for the detection of *Y*. *pestis* at a low concentration of 10^2^ CFU/100 mg soil.

**Table 3:** Comparison of the ddPCR assay and other PCR-based assays for *Y*. *pestis*.

PCR-based assays for *Y*. *pestis*	Detection sensitivity (CFU/sample)			Sample type (volume/weight)	Ref.
	chromosome	*caf1*	*pla*		
^**a**^ **FilmArray Biothreat Panel test**	Not tested	~10	~10	mouse blood (100 μL)	[10, 11]
^**b**^ **GeneXpert TAP assay**	Not tested	Not tested	4.5	human blood (1 mL)	[9]
^**b**^ **TAC assay**	4×10^3^	4×10^3^	4×10^2^	mouse blood (250 μL)	[11]
**ddPCR assay**	10^3^	10^3^	~10	mouse liver (20 mg)	This study
**Singleplex qPCR assay**	10^4^	10^4^	Not tested	Soil (200 mg)	[26]
^**c**^ **ddPCR assay**	10^2^	Not tested	Not tested	Soil (100 mg)	[23]
**ddPCR assay**	10^3^	10^2^	~10	Soil (100 mg)	This study

^a^ The detection targets include *Y*. *pestis* and 15 other biothreat pathogens.

^b^ The detection targets include *Y*. *pestis*, *F*. *tularensis*, and *B*. *anthracis*.

^c^ The detection targets include *Y*. *pestis* and four other biothreat pathogens.

In this study, the performance of the ddPCR assay was evaluated using *Y*. *pestis*-spiked environmental soil and mouse liver samples, with pure culture samples as comparisons. The detection sensitivity was not significantly interfered with by the soil matrix (10^2^ CFU/sample) but was 10-fold interfered with by the liver tissue matrix (10^3^ CFU/sample). This result is similar to our previous study [18], which showed that lung tissue samples had a greater influence on PCR sensitivity than soil samples. In fact, the detection sensitivity of 10^2^ CFU/sample is equivalent to approximately 4 CFU/reaction, as 2 μL of the 50 μL extracted DNA was used as the template. For such a small amount of DNA, a slight impact of residual inhibitors in the reaction is considered acceptable. Despite the interference, our method still displayed higher sensitivity than qPCR when detecting *Y*. *pestis* in these complex samples.

There are several limitations to our method. First, the detection primers and probes of the *pla* gene used in this study need further improvement in specificity [32]; other genes (such as *pst*) on the pPCP1 plasmid are an alternative choice to increase the specificity [33]. Second, *Y*. *pestis* strains containing neither the pMT1 nor the pPCP1 plasmid may exist in nature. For these strains, our approach would not work because only one chromosomal target gene can be detected. Therefore, at least two different specific genes on the chromosome should be included in future ddPCR assays, such as *ypo2486* [15] and *ypo0392* [34]. Moreover, while ddPCR presents higher sensitivity, it involves a more time-consuming process compared to qPCR approach, due to the inclusion of additional steps such as droplet formation and individual droplet signal analysis. Lastly, we estimated the direct cost of each DNA detection (for the amplification step only) in our laboratory: about 3 US dollars per qPCR test (overall cost for three gene targets) and 5 US dollars per ddPCR test. Therefore, reducing ddPCR assay costs and improving its availability in resource-limited environments are still warranted.

In conclusion, we developed a highly sensitive and specific ddPCR assay to detect *Y*. *pestis* DNA in this study. This approach can be applied to detect low concentrations of *Y*. *pestis* DNA from soil and animal tissue samples, which is of great potential for improving the detection rate of *Y*. *pestis* in plague surveillance and facilitating the early diagnosis of plague cases.

## Supporting information

S1 Fig The qPCR performance in detecting *Y*. *pestis* DNA from simulated samples. (PDF)

S1 Table Detection results of the blank samples (ddH_2_O) and calculation of LoBs. (PDF)

S2 Table Performance of the assay in detecting *Y*. *pestis* DNA from soil samples. (PDF)

S3 Table Performance of the assay in detecting *Y*. *pestis* DNA from liver samples. (PDF)

S1 Data Excel spreadsheet containing, in separate sheets, the underlying numerical data for Figs 1–4. (XLSX)
