# Supplementary figures and images for: Development and evaluation of a multi-target droplet digital PCR assay for highly sensitive and specific detection of Yersinia pestis
Source: PLoS Negl Trop Dis. 2024 May 3;18(5):e0012167. doi: 10.1371/journal.pntd.0012167 (PMC11095742; doi:10.1371/journal.pntd.0012167)

**S1 Fig. The qPCR performance in detecting *Y. pestis* DNA from simulated samples.**

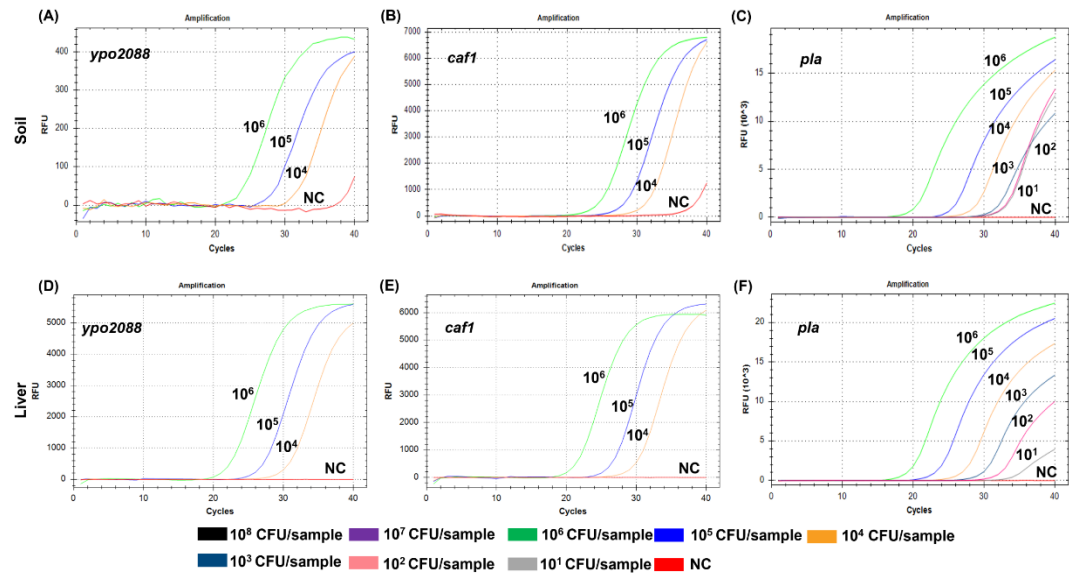

Supplement: S1 Fig — (PDF) [file pntd.0012167.s001.pdf]
