# Supplementary material for: Development and evaluation of a multi-target droplet digital PCR assay for highly sensitive and specific detection of Yersinia pestis
Source: PLoS Negl Trop Dis. 2024 May 3;18(5):e0012167. doi: 10.1371/journal.pntd.0012167 (PMC11095742; doi:10.1371/journal.pntd.0012167)
Supplement: S1 Table — (PDF) [file pntd.0012167.s002.pdf]

S1 Table. Detection results of the blank samples (ddH_2O) and calculation of LoBs.

Number	Blank results (copies/μL)		
	ypo2088	*caf1*	*pla*
N_1	0	0	0
N_2	0.06	0	0
N_3	0.07	0	0
N_4	0.09	0	0
N_5	0.09	0.07	0
N_6	0.10	0.07	0
N_7	0.11	0.08	0
N_8	0.11	0.09	0
N_9	0.11	0.1	0
N_{10}	0.11	0.11	0
N_{11}	0.12	0.11	0
N_{12}	0.14	0.12	0
N_{13}	0.16	0.12	0
N_{14}	0.17	0.15	0
N_{15}	0.17	0.16	0
N_{16}	0.19	0.17	0
N_{17}	0.20	0.17	0
N_{18}	0.22	0.18	0.08
N_{19}	0.24	0.18	0.08
N_{20}	0.30	0.18	0.09
N_{21}	0.31	0.19	0.09
N_{22}	0.31	0.20	0.09
N_{23}	0.32	0.22	0.09
N_{24}	0.33	0.23	0.09
N_{25}	0.39	0.24	0.09
N_{26}	0.39	0.24	0.10
N_{27}	0.40	0.25	0.10
N_{28}	0.42	0.26	0.10
N_{29}	0.46	0.27	0.10
N_{30}	0.47	0.28	0.11
N_{31}	0.52	0.30	0.11
N_{32}	0.54	0.30	0.11
N_{33}	0.58	0.32	0.11
N_{34}	0.61	0.32	0.35
N_{35}	0.63	0.33	0.66
[a] **LoB**	[b] 0.57	[c] 0.32	[d] 0.11

[a] As per the CLSI EP17-A2 guideline, Rank Position=0.5+*35*• 0.95=33.75

[b] LoB (*ypo2088*) = N_{33} + 0.75• (N_{34} - N_{33}) = 0.58+0.75• (0.61-0.58) = 0.57 copies/μL

[c] LoB (*caf1*) = N_{33} + 0.75• (N_{34} -N_{33}) = 0.32+0.75• (0.32-0.32) = 0.32 copies/μL

[d] LoB (*pla*) = N_{33} + 0.75• (N_{34}- N_{33}) = 0.11+0.75• (0.11-0.11) = 0.11 copies/μL
