# Supplementary material for: Development and evaluation of a multi-target droplet digital PCR assay for highly sensitive and specific detection of Yersinia pestis
Source: PLoS Negl Trop Dis. 2024 May 3;18(5):e0012167. doi: 10.1371/journal.pntd.0012167 (PMC11095742; doi:10.1371/journal.pntd.0012167)
Supplement: S2 Table — (PDF) [file pntd.0012167.s003.pdf]

**S3 Table. Performance of the assay in detecting *Y. pestis* DNA from soil samples.**

| Detection method | Groups | ddPCR | | qPCR | | Total |
|---|---|---|---|---|---|---|
| | | Positive | Negative | Positive | Negative | |
| Plate counting confirmed | **Positive** | 28 | 7 | 20 | 15 | 35 |
| | **Negative** | 1 | 29 | 1 | 29 | 30 |
| | **Total** | 29 | 36 | 21 | 44 | 65 |
