# Supplementary material for: Development and evaluation of a multi-target droplet digital PCR assay for highly sensitive and specific detection of Yersinia pestis
Source: PLoS Negl Trop Dis. 2024 May 3;18(5):e0012167. doi: 10.1371/journal.pntd.0012167 (PMC11095742; doi:10.1371/journal.pntd.0012167)
Supplement: S3 Table — (PDF) [file pntd.0012167.s004.pdf]

S4 Table. Performance of the assay in detecting *Y. pestis* DNA from liver samples.

Detection method	Groups	ddPCR		qPCR		Total
		**Positive**	**Negative**	**Positive**	**Negative**	
**Plate counting confirmed**	**Positive**	22	13	20	15	35
	**Negative**	1	29	1	29	30
	**Total**	23	42	21	44	65
